# An Update in Drug-Induced Thrombotic Microangiopathy

**DOI:** 10.3389/fmed.2020.00212

**Published:** 2020-05-22

**Authors:** Thomas Chatzikonstantinou, Maria Gavriilaki, Achilles Anagnostopoulos, Eleni Gavriilaki

**Affiliations:** ^1^BMT Unit, Hematology Department, G Papanicolaou Hospital, Thessaloniki, Greece; ^2^Laboratory of Clinical Neurophysiology, AHEPA Hospital, Aristotle University of Thessaloniki, Thessaloniki, Greece

**Keywords:** thrombotic microangiopathy, drug, hematology, oncology, neurology

## Introduction

Drug-induced thrombotic microangiopathy (DITMA) is a life-threatening complication that is often under-recognized and under-reported (1). Despite recent systematic reviews published in 2015 (2) and 2018 (3), the list of drugs implicated in TMA continues to expand (4–9). In addition, novel reports have unraveled potential new mechanisms that might contribute to a targeted therapy of this syndrome. In this opinion article, we aimed to summarize recent data on DITMA, categorizing drugs based on mechanisms of actions and specialties.

## Mechanisms of Action

Two decades ago, George et al. introduced the term “drug-induced thrombocytopenia.” Clinically based criteria were proposed and levels of evidence were stratified in order to solidify a definite, propable, possible, or an unlikely causal relationship between a drug and thrombocytopenia (10). Although the mechanisms of endothelial injury during DITMA still remain unknown; immune-mediated mechanisms or dose-dependent and cumulative toxicity are implied (11). The hypothesis is based on the observation of the timing of TMA occurrence, the pattern of disease, the exclusion of a better explanation thorough investigation. DITMA suspicion is ampilified by TMA resolution when the drug is withdrawn or recurrent endothelial injury during re-exposure to the drug.

**Figure 1:**
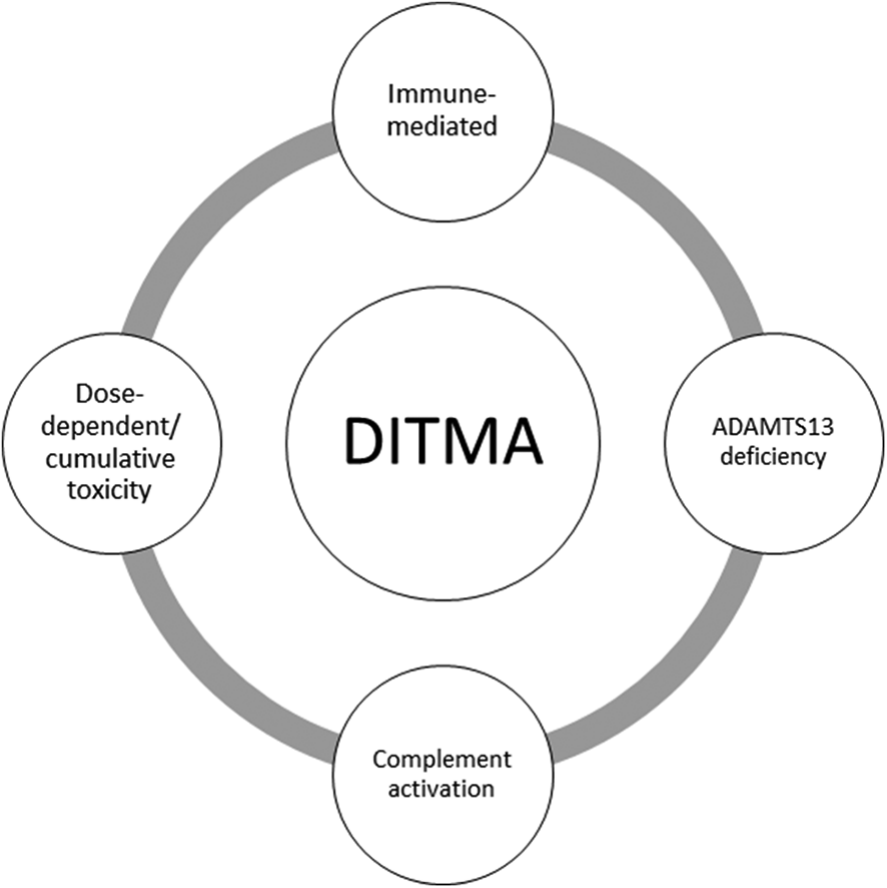
Summary of postulated mechanisms in drug-induced thrombotic microangiopathy (DITMA). ADAMTS13: a disintegrin and metalloproteinase with a thrombospondin type 1 motif, member 13.

During the last decade, laboratory criteria have been added to support the causal relationship between a drug and TMA (2). Some examples of drugs in which antibody mediated DITMA has been confirmed with identification of drug-dependent antibodies to platelets or other cells as the pathophysiologic mechanism of TMA are quinine, oxaliplatin, and vancomycin (12). On the other hand, the dose-dependent and cumulative toxicity model seems to fit for opana's abuse, bevacizumab, levofloxacin, alemtuzumab, and interferon's cases of DITMA (9, 13–15). It is important to exclude any other diagnosis before attributing TMA to a drug. For example, in some cases such as these of ipilimumab, pazopanib, ustekinumab, and golimumab severe ADAMTS13 deficiency was found, plasma exchange was effective and no drug-dependent antibody inhibition of ADAMTS13 activity was reported, making drug-indused causal relationship unlikely (7, 16–18). Figure 1 summarizes postulated mechanisms in DITMA.

## Hematology/Oncology

DITMA is caused by various drugs used in Hematology and Oncology.

### Chemotherapy

Chemotherapeutic agents were the first to be implicated in causing DITMA. Mitomycin and gemcitabine have numerous reports of dose-related DITMA, while one report describes an immune-DITMA as a result of gemcitabine administration (19–26). Despite their use as a combination with other drugs, which makes the direct causal relationship difficult in some cases, many well-described cases support a clear-cut association (21, 27, 28).

Three reports have described DITMA caused by pentostatin, a purine analog used in lymphoproliferative diseases (29). Docetaxel and vincristine have also been reported to induce TMA (30, 31). Oxaliplatin has been implicated as a cause of DITMA in a review by Al-Nouri et al. (2), although the authors of the original report described gemcitabine as the causative factor (32). Renal-limited TMA has been reported in three patients treated with pegylated liposomal doxorubicin (33) and in one patient receiving treatment with a short interfering RNA targeted against Myc (DCR-MYC) (5).

### Multiagent Chemotherapy

Drug-induced TMA has been reported in children with acute lymphoblastic leukemia (34, 35) and adults with solid tumors (36, 37), receiving multiagent chemotherapy. Jodele et al. described 13 patients developing TMA after high-dose chemotherapy and autologous stem cell transplantation for neuroblastoma (12 patients receiving carboplatin/etoposide/melphalan and one cyclophosphamide/thiotepa) (38). Finally, a high incidence of TMA was observed in melanoma patients receiving a lymphodepleting preparative chemotherapy regimen with total body irradiation (TBI) prior to autologous T cell therapy (39). In all cases, the co-administration of multiple drugs hinders the identification of the causative agent.

### Proteasome Inhibitors

Proteasome inhibitors are mainly used in multiple myeloma treatment and have been associated with DITMA (bortezomib, carfilzomib, ixazomib). The majority of reports have implicated bortezomib and cafilzomib (3, 40). Recent reports also support a causal association of ixazomib with DITMA (41–43). Some authors report successful treatment of carfilzomib-induced TMA with eculizumab (44, 45).

### VEGF, Kinase and Immune Checkpoint Inhibitors

Vascular endothelial growth factor (VEGF) inhibitors are used for the treatment of various malignancies. Many cases reported DITMA as a result of bevacizumab use, a humanized monoclonal antibody directed against VEGF (46–49). In some, treatment with eculizumab was successful (50). Ramucirumab, anti-VEGF receptor 2 monoclonal antibody, and cetuximab, a monoclonal antibody against epidermal growth factor receptor (EGFR), are also implicated in causing renal-limited TMA (51, 52).

Tyrosine kinase inhibitors (TKIs) are effective in the treatment of hematologic malignancies and solid tumors. Sunitinib is a small-molecule TKI that targets VEGFR-2 and PDGFR-b. Imatinib and ponatinib are small-molecule BCR-ABL TKIs, used mainly in the treatment of chronic myeloid leukemia. Palbociclib inhibits the cyclin-dependent kinases CDK4 and CDK6. Cases reporting a link between the aforementioned TKIs and drug-induced TMA have been described in literature (6, 53–56).

Two cases of TMA, one in a patient receiving the immune checkpoint inhibitor ipilimumab, and one in a patient treated with multi-targeted receptor tyrosine kinase inhibitor pazopanib have also been reported. However, these reports differ from other DITMAs, due to the severe ADAMTS13 deficiency (7, 16).

### Calcineurin and mTOR Inhibitors

Numerous reports implicate cyclosporine and tacrolimus in causing dose-dependent TMA (2). Most of these reports described calcineurin inhibitor-induced TMA in patients that have undergone hematopoeitic stem cell or solid organ transplantation (57). Calcineurin inhibitor-induced TMA mostly affects the kidneys (58, 59). The inhibitors of the mechanistic target of rapamycin (mTOR), can also cause DITMA, most frequently associated with sirolimus or tacrolimus administration, than everolimus (60–62). Successful treatment with complement inhibition has been described in several patients, since this condition along with TA-TMA has been considered to resemble atypical hemolytic uremic syndrome (aHUS) (63, 64).

## Monoclonal Antibodies

The first reported case of monoclonal antibody-induced TMA described a patient treated with anti-T cell monoclonal antibody muromonab-CD3 (OKT3) (65). Emicizumab, a monoclonal antibody used in Hemophilia A, co-administered with high doses of activated prothrombin complex concentrate (aPCC) has been linked with TMA in three patients (66). Discontinuation of aPCC resulted in resolution of TMA, highlighting the fact that emicizumab monotherapy may not be sufficient to cause DITMA.

Various monoclonal antibodies against tumor necrosis factor alpha (TNF-a), such as adalimumab, golimumab, and certolizumab pegol have been reported to cause TMA in a few cases (17, 18, 67). Another report describes a patient with psoriasis developing TMA after treatment with methotrexate and ustekinumab, a monoclonal antibody that blocks interleukin (IL)-12 and IL-23 (68). However, in the cases where ustekinumab and golimumab were suspected to be the causative factor of DITMA, the authors reported low levels of ADAMS13 (<5%) and an initial response to plasma exchange, making DITMA diagnosis unlikely (17, 18).

In phase 1 study, moxetumomab pasudotox, an anti-CD22 immunotoxin used in the treatment of childhood acute lymphoblastic leukemia, caused TMA in 13% of patients. In the majority of cases, TMA resolved with drug discontinuation (69).

## Opioids and Other Drugs of Abuse

Intravenous use of the extended-release opioid oxymorphone and oxycodone tablets reformulated with polyethylene oxide (PEO) have been reported to cause DITMA in many patients (3). Subsequently, intravenously administrated high molecular PEO was determined as the causative factor (15). Cocaine and ecstasy have caused DITMA in recreational users (70–72).

## Neurology

One of the biggest challenges in neurology is the lack of disease-specific drugs that contributes to the increasing global burden of neurological disorders (73). Traditionally, epilepsy benefited from a wide variety of available medicines but during the last decade numerus drugs were introduced at multiple sclerosis (MS) treatment raising long-term safety considerations (74, 75). Until 2018, interferon beta 1-a and 1-b, disease modifying treatments (DMTs) of MS and anticonvulsive valproic acid were the only neurologic drugs associated with thrombotic microangiopathy (3).

Recently, alemtuzumab; which was approved by US Food and Drug Administration (FDA) for treatment of relapsing-remitting MS (RRMS) at 2014; was associated for the first time with DITMA (8, 9). Administration of alemtuzumab was known to rarely cause severe renal adverse effects (76). Nevertheless, in that case report the causal relationship of alemtuzumab with TMA is supported by the fact that (a) symptoms started immediately after the first infusion and (b) the patient did not respond to plasma exchange (9). Another DMT, fingolimod was linked with TMA in an induced-malignant hypertension animal model; in contrast with control group in which fingolimod was not administrated (77). Interferon (IFN) has also been correlated with TMA with a dose-dependent manner (14). Further studies confirm that TMA is a severe complication of IFN-beta RRMS treatment. Lately, five patients were reported to have IFN-induced TMA following long-term treatment (78–80). Interestingly, renal function of three patients improved only after administration of eculizumab, not after withdrawal of IFN (80). Clinical translation of those studies raises awareness of neurologists for early recognition and management of TMA when prescribing DMTs.

## Infectious Diseases

An infection can be caused by a variety of organisms such as bacteria, viruses, parasites or fungi. Many anti-infectives agents have been associated with DITMA in the past (2); quinine, the treatment of malaria, was the most commonly reported (81).

Novel studies implicate a number of different drugs in causing DITMA. First of all, a case report incriminates hydroxychloroquine, a synthetic derivative of quinine used for rheumatoid arthritis and systemic lupus erythematosus, as a possible cause of thrombotic thrombocytopenia purpura (TTP) (82). Disease progression was detrimental and patient died in spite of drug withdrawal and plasma exchange. Moreover, for the first time an antiretroviral treatment of human immunodeficiency virus consisting of tenofovir/emtricitabine was found to have a causality relationship with immune TTP (83). After the cessation of the drug and the initiation of corticosteroids and azathioprine the patient recovered. Last but not least, several antibiotics such as ciprofloxacin, penicillin, and metronidazole were reported with probable evidence to cause DITMA (2). A new case report implicates again ciprofloxacin in drug-induced TTP which resolved completely with plasma exchange (84). Another report, identified a highly effective and frequently prescribed fluoroquinolone, levofloxacin as a new potential suspect for DITMA (13). This case report described two patients who developed microangiopathic hemolysis and thrombocytopenia following levofloxacin treatment of respiratory tract infections. Both cases resolved after drug cessation; the first patient received also therapeutic plasma exchange. In conclusion, a wide variety of anti-infectives agents have been scarcely correlated with DITMA and unfortunately, no one could predict or prevent its appearance; hence, it is of paramount importance to be aware of that possibility in order to start the appropriate treatment promptly.

## Therapeutic Potentials

The only proven intervention in the management of DITMA is discontinuation of the offending agent. Plasma exchange and immunosuppressive therapy may be a reasonable treatment option, especially when the diagnosis is uncertain. Although rarely described in DITMA, patients with severe ADAMTS13 deficiency respond to plasma exchange (7, 16–18, 82–84). However, these reports should be interpreted with caution, since severe ADAMTS13 deficiency indicates TTP as a more likely diagnosis. In true DITMA, plasma exchange is ineffective (85). On the other hand, numerous reports have now confirmed that complement inhibition with eculizumab shows efficacy in DITMA (22, 26, 44, 45, 50, 63, 80). Eculizumab is a first-in-class monoclonal antibody that blocks terminal complement activation with proven safety and efficacy in complement-mediated TMAs (86). Based on current literature, we would consider eculizumab administration in three instances: in patients with non-immune DITMA, in those who deteriorate despite discontinuation of the implicated drug and supportive care, and finally, in patients at risk of kidney failure (87).

## Conclusions and Future Perspectives

Our state-of-the-art report categorizes drugs that have been associated with DITMA, summarized in Table 1. It also emphasizes on unique presentations and characteristics of DITMA, that require increased awareness by treating physicians of relevant specialties. Hematologists are largely involved in the administration of the majority of these drugs, along with other internal medicine specialties. Since many patients have presented with renal-limited complications, the role of nephrologists is also important. Therefore, our report highlights an unmet clinical need of increased recognition and better understanding of DITMA by treating physicians across different specialties.

**Table 1:** Summary of drugs involved in DITMA.

**Drug**	**Type**	**Specialty**
Docetaxel Doxorubicin DCR-MYC Gemcitabine Oxaliplatin Pentostatin Vincristine	Chemotherapy	Hematology/Oncology
Carboplatin + Etoposide + Melphalan Cyclophosphamide + Thiotepa	Multiagent chemotherapy	
Bortezomib Carfilzomib Ixazomib	Proteasome inhibitors	
Bevacizumab Ramucirumab Cetuximab Imatinib Ipilimumab Pazopanib Ponatinib Palbociclib Ruxolitinib Sunitinib	VEGF, kinase and immune checkpoint inhibitors	
Cyclosporine Rapamycin Tacrolimus	Calcineurin and mTOR inhibitors	
Adalimumab Certolizumab pegol Emicizumab + aPCC Golimumab OKT3 Ustekinumab Moxetumomab pasudotox	Monoclonal antibodies	Hematology/Oncology/ Rheumatology
Cocaine/ Ecstasy Oxymorphone/Oxycodone Polyethylene oxide (PEO)	Opioids / Drugs of abuse	Toxicology
Interferon beta 1-a /1-b Alemtuzumab Fingolimod	Disease modifying treatments for Multiple Sclerosis	Neurology
Valproic acid	Anticonvulsive	
Tenofovir/Emtricitabine	Anti-infectives	Infectious diseases
Quinine/Hydroxychloroquine	Antimalarials	
Ciprofloxacin Fluoroquinolone Metronidazole Penicillin	Antibiotics	

Except for expanding the list of drugs associated with DITMA, future reports need to take into account potential mechanisms. Identification of underlying etiology is of utmost importance for proper management. Further mechanistic studies need to identify the drugs or pathways involved in complement activation in order to early select patients that would benefit from complement inhibition.

## Author Contributions

EG conceived the manuscript concept. TC and MG wrote the manuscript. AA and EG edited and approved the final manuscript.

## Conflict of Interest

The authors declare that the research was conducted in the absence of any commercial or financial relationships that could be construed as a potential conflict of interest.
